# Emotion Regulation Ability and Resilience in a Sample of Adolescents from a Suburban Area

**DOI:** 10.3389/fpsyg.2017.01980

**Published:** 2017-11-13

**Authors:** José M. Mestre, Juan M. Núñez-Lozano, Rocío Gómez-Molinero, Antonio Zayas, Rocío Guil

**Affiliations:** ^1^Instituto de Desarrollo Social y Sostenible (INDESS), University of Cádiz, Cádiz, Spain; ^2^Centro de Apoyo a la Formación e Innovación Educativa de Irún, Gipuzkoa, Spain

**Keywords:** emotion regulation ability (ERA), cognitive regulation strategies, resilience, adolescence, adaptation

## Abstract

Earlier research has identified a remarkable number of related factors to resilience during adolescence. Historically, theoretical treatments of resilience have been focused almost exclusively on psychosocial levels of analysis to derive explanatory models. However, there is insufficient understanding of the role of emotion regulation explaining competent functioning despite the experience of adversity (resilience), especially during adolescence. This study explores the relationship between both, emotional regulation abilities and strategies, and resilience in a sample of adolescents from suburbs high-schools (Jerez de la Frontera, Spain). The study also examines how using different emotional regulation strategies may help the development of resilience levels at this stage. Participants of the study were 164 adolescents ranging from 13 to 16 years old (*M* = 13.98*; SD* = 0.66). Emotion regulation was measured using the Cognitive Emotional Regulation Questionnaire (CERQ, Garnefski et al., 2001), and sections D and H of Mayer-Salovey-Caruso Emotional Intelligence Test, a performance test (Emotion Regulation Ability sections, MSCEIT, Spanish version, Mayer et al., 2003). Resilience was evaluated with ERE (Educative Resilience Scale for children and adolescents, Saavedra and Castro, 2009). Verbal Intelligence (Yuste, 1997) and personality traits (Cattell and Cattell, 1986) were assessed as two independent variables. Results supported the idea that emotion regulation ability (MSCEIT, D and H sections, Extremera et al., 2006) is a significant predictor of adolescents' resilience. Moreover, cognitive regulation strategies, such as positive reappraisal, predicted perceived resilience among students. Sociability (A factor of HSPQ, sociability) also correlated with resilience levels. Hence, these results are promising, implying that emotion regulation ability may act as a helpful tool preventing adolescents from irrational risky behaviors, commonly assumed at this developmental stage.

## Introduction

Many changes and vulnerabilities affect adolescents at this life stage. Moving from primary school to high-school, unfavorable socioeconomic conditions, and personal attributes may favor risky behaviors that often lead to school failures. Risk factors understood as individual, school, peer, family, and community influences might increase the probability that a child will experience, maintain, or exacerbate, both social and mental problems (Shumow et al., 1999; Jenson and Fraser, 2005). According to Kurian (2012), low socioeconomic condition influenced teenagers' psychological growth and development. The fact that some adolescents get over these risk factors and triumph in the academic and social context depends on their resilience development (Wang et al., 1995; Waxman et al., 2003).

### Resilience: is a fixed or ongoing term?

Defining resilience has been controversial, and still, there is not enough consensus regarding a unified description of the construct (see Afifi and MacMillan, 2011; Lee et al., 2013). According to Lee et al. (2013), resilience definitions can be grouped into two different perspectives: resilience as a *trait* vs. resilience as a *developmental process*.

The first group of definitions —resilience as a trait- stated that resilience is the ability to “bounce back.” This approach is in line with the physical sciences, which postulated that resilience is a property that allows materials to assume their original shape after being bent or stretched (Dyer and McGuinness, 1996; Southwick and Charney, 2012). Particularly within this view, Block and colleagues introduced the concept of *ego-resilience* as “the dynamic capacity to modify his or her model level of ego-control, in either direction, as a function of the demand characteristics of the environmental context” (Block and Kremen, 1996, p. 48). However, these definitions implied that resilience is fixed and stable, and thus unable to explain the adaptation between individuals and the environment (Lee et al., 2012). Moreover, the concept of ego-resilience did not infer the presence of adverse situations (Luthar et al., 2000).

Conversely, the second group of authors stated that resilience is a dynamic process by which individuals successfully adapt within the context of significant adversity (Luthar et al., 2000; Connor and Davidson, 2003; Masten and Obradović, 2006; Lee et al., 2013). Within this view, resilience is changeable over time and influenced by protective factors (Dyer and McGuinness, 1996; Connor and Davidson, 2003; Lee et al., 2013). According to Dyer and McGuinness (1996), protective factors are abilities and skills (competencies) needed to build resilience. The authors identified three types of competencies: individual, interpersonal, and familiar. Thus, resilience is characterized as a developmental process in which the environment is crucial determining personal resilience (Brownlee et al., 2013). Accordingly, a group of APA's experts defined resilience as a “process of adapting well in the face of adversity, trauma, tragedy, threats or even significant sources of threat” (American Psychological Association, 2017). We agree with this second view of resilience as a dynamic process stating that in fact, resilience can be viewed as the successful adaptation and development to stressful situations (Masten, 2001; Tugade and Fredrickson, 2004).

### Resilience and emotional-cognitive abilities

Reviewing the literature, we have found studies that show the importance of *cognitive and emotional abilities* predicting resilience. For example, Artuch-Garde et al. (2017) exposed in their cross-sectional research that the ability to self-regulate behavior is associated with high levels of resilience in high-school students. Likewise, positive emotions appeared to aid resilient people to deal with daily strain (Tugade and Fredrickson, 2004; Ong et al., 2006). Gloria et al. (2013) developed a cross-sectional study demonstrating that positive affectivity strongly predicts resilience among public school teachers. In particular, this research has revealed that positive affectivity increases one's cognitive resilience levels, allowing individuals to effectively deal with adversity and stress (Gloria et al., 2013). In later ages, emotional-cognitive abilities might have an important role promoting also both personal and social functioning. For example, implementing an emotional-based intervention in a sample of Alzheimer's patients increases motivational process to reduce apathy among patients that were randomly assigned to the brief emotional intervention (see Di Domenico et al., 2016).

According to the emotion regulation (ER) meta-analysis conducted by Naragon-Gainey et al. one of the ER's theoretical models is the Ability-Based ER model—besides Strategy-Based ER Model and Temporal Process ER Models (Naragon-Gainey et al., 2017). The Ability-Based ER model “are primarily organized around dispositional abilities believed to facilitate healthy emotion regulation” (Naragon-Gainey et al., 2017 p. 386). To assess these dispositional abilities to solve risky emotional situations, i.e., emotional intelligence as ability, we believe that is more appropriate the use performance measures rather than self-report instruments (see MacCann et al., 2014; Mayer et al., 2016; Mestre et al., 2016). However, after a literature review concerning ERA and resilience, authors do not consider performance measures in the research of the role of ERA in perceived resilience among adolescents.

Resilience has also been associated with *coping strategies* (Beasley et al., 2003; Campbell-Sills et al., 2006; Min et al., 2013; Lee et al., 2017). Lazarus and Folkman (1984) have classically defined cognitive coping strategies as “constantly changing cognitive and behavioral efforts to manage specific external and internal demands that are appraised as taxing or exceeding the resources of a person” (p. 141). According to this definition, coping strategies present two main functions: problem-solving focus coping strategies, or acting on the stressor; and emotion-focus coping strategies, or managing emotions provoked by the stressor (Garnefski et al., 2001). Some authors have supported this assumption stating that problem-solving strategies lead to a better adaptation to adversity while emotion-focus strategies drive to a worst adjustment (Beasley et al., 2003; Campbell-Sills et al., 2006). However, recently, Lee et al. (2017) found out that emotion-focus coping strategy was also determinant enhancing a resilient outcome. In particular, these authors carried out a cross-sectional study and demonstrated that resilient adolescents engage in both strategies simultaneously, emotion-focus and problem-solving focus. Nevertheless, Garnefski et al. (2001) established a new division of coping strategies, behavioral and cognitive, arguing that cognitive coping precede behavioral coping strategies. In other words, whether this process is conscious or unconscious, when a potentially adverse situation arrives, individuals first think and then act. Thus, teaching people how to react in the face of adversity, focusing in their cognitions, may favor a resilient response (Garnefski et al., 2001). Moreover, they also pointed out the simplistic classification made by Monat and Lazarus (1991), stressing people engage in many more coping strategies than emotion or problem-solving focus. Notably, they proposed a set of nine coping strategies: self-blame, acceptance, focus on thought/rumination, positive refocusing, refocus on planning, positive reappraisal, putting into perspective, catastrophizing, and blaming others (Garnefski et al., 2001). Following this statement, Min et al. (2013) examined the relationship between cognitive coping strategies and resilience among depressive and/or anxiety disorder patients. This cross-sectional study suggested that engaging in adaptive strategies, such as positive reappraisal and refocus on planning, were the two most significant predictors of resilience among adult population. However, many of the research conducted so far, concerning coping strategies and resilience, have been concentrated on adulthood, and little is known about the role played by these cognitive strategies during adolescence.

Because stressful events have by nature an emotional component, people's ability to *manage emotions* may be another critical factor determining resilience (Caston and Mauss, 2011). Indeed, school life is full of emotional stimuli (even stress), which affect students' motivations and academic achievement (Pekrun et al., 2002; Lopes et al., 2012). Emotion research has demonstrated the importance of emotion regulation in adaptation, cognition, well-being, attention, and social interaction (f. i., Peña-Sarrionandia et al., 2015; Mayer et al., 2016). In fact, emotional dysregulation can undermine decision-making processes, increase anxiety, and produce a lack of social competence (Loewenstein and Lerner, 2003; Wills et al., 2016; Hartman et al., 2017). Emotion regulation has been defined as “all the extrinsic and intrinsic processes responsible for monitoring, evaluating and modifying emotional reactions, especially their intensive and temporal features, to accomplish one's goals” (Thompson, 1994, pp. 27–28). In this study, we focused on adolescents' ability to manage own and others' emotions to adapt in the face of adversity successfully. Thus, we conceptualized emotion regulation ability (ERA) following Mayer and Salovey's emotional intelligence theory (Mayer and Salovey, 1997; Mayer et al., 2016). According to Mayer and colleagues, emotional intelligence is defined as the ability to problem-solving with and about emotions (Salovey and Mayer, 1990; Mayer and Salovey, 1997; Mayer et al., 2003). The concept comprises four interrelated abilities involved in the intake of emotional information: perceiving emotions, using emotions to facilitate thought, understanding emotions, and regulating own and other's emotions (Lopes et al., 2005, 2012). As we mentioned above, only the fourth branch of emotional intelligence, management of emotions, was considered for this research. Hence, ERA constitutes the ability to differentiate, label, and display both, own and others' emotions as well as using appropriate strategies to modify own and other's feelings. In this regard, ERA plays a key role favoring adolescents mental health and school adaptation (Brackett and Salovey, 2006; Mestre and Guil, 2006; Rivers et al., 2012; Sánchez-Álvarez et al., 2015; Fernandez-Berrocal and Extremera, 2016).

### Resilience, personality traits, and IQ

Other non-emotional psychological factors have also been considered regarding resilience research. For example, personal characteristics, such as self-concept and intelligence, appeared as the most significant predictors of resilience during adolescence (Garza et al., 2014). Other studies have demonstrated that other personality factors such as extraversion significantly predicted resilience (Campbell-Sills et al., 2006; Hsieh et al., 2016; de las Olas Palma-García and Hombrados-Mendieta, 2017). Following the Five-factor model of personality, Friborg et al. (2005), in a sample of 482 applicants for a military college, found that resilience factors were positively correlated with a well adjusted personality profile, which included “personal strength,” “social competence,” “structured style,” “family cohesion,” and “social resources.” However, resilience was unrelated to cognitive abilities. Based on the view of resilience as a dynamic process, Campbell-Sills et al. (2006) pointed out that resilience was negatively associated with neuroticism, and positively related to extraversion and conscientiousness.

Concerning the relationship between resilience and personality among adolescents, we have found discrepancies in the literature. For example, some studies used personality instruments that are not specifically designed for adolescents, while others show correlations among very different personality factors. Moreover, the resilience approach (fixed vs. ongoing) may also influence the outcome variance founded.

Regarding IQ and resilience, conceptually, some degree of connection between both variables is expected. According to the idea that adaptability is an indicator of efficient functioning of underlying intellective components, Block and Kremen (1996) investigated the expected relationship between ego-resilience and IQ. The authors, based on a trait resilience view, found out a relationship between resilience and IQ, only significant for the female sample (*R* = 0.10 females, *R* = 0.31 males), measured in individuals at the age of 18. Anyhow, they called for further studies to understand the role of IQ on resilience. According to the view of resilience as a developmental factor, we believe that IQ measures should be more focused on crystallized IQ (Gc)—f. i., verbal intelligence- than on fluid IQ (Gf). In this sense, Gc is more related to external experiences than Gf and has stronger connections to emotional-cognitive abilities as emotional intelligence (Mestre et al., 2016). Friborg et al. (2005) developed a study with 411 applicants for military college. Participants had to complete a resilience, personality, and IQ questionnaire. Confirmatory factor analyses confirmed the fit of the five-factor model, however, authors found anunrelated relationship between cognitive abilities and resilience. Nonetheless, this study was applied to young adults instead of adolescents. Contrary to their expectations, IQ had an “insignificant and negligible” relationship with resilience (Friborg et al., 2005, p. 38). Even though the efforts made up to date, it is still unclear the role of IQ on resilience perception.

### Resilience at adolescence stage: the role of emotional-cognitive abilities

Although literature has been interested in the role of resilience during adolescence (Compas et al., 1995; Hunter and Chandler, 1999; Anthony et al., 2009; Skrove et al., 2013; Newsome and Sullivan, 2014), little is known about the role played by emotional-cognitive abilities in the development of resilience. Because resilience allows individuals to cope with adversity, this concept may be relevant to adolescents dealing with school adaptation. Due to the lack of studies concerning the cognitive and emotional processes underlying resilience at this life stage, the overall objective of this research was to explore the relationship between both ERA and cognitive-emotional strategies, and perceived resilience among adolescents. Particularly, including other predictors as personality and verbal intelligence, we proposed that adolescents high on ERA would present higher levels of perceived resilience. Moreover, we expected that appraisal cognitive-emotional regulation strategies would also lead to higher levels of resilience. The study highlighted the importance of building resilience, based on the development of cognitive and emotional abilities, in adolescents under a risk context.

## Method

### Participants and procedure

The sample comprised 164 Spanish adolescents from eighth grade of middle school and ninth high school (second and third of compulsory schooling in Spain). Participants were selected by quota sampling from a suburban area of schools in Jerez de la Frontera, Spain. Participants average age was 13.98 (range = 13–16, *SD* = 0.66). Adolescents were nearly equally divided by gender (53.9% male). Participants had to complete a questionnaire with all the scales presented at the same time during class time. Participation was anonymous and volunteer, and data collection was accomplished by following the ethical guidelines applicable to people under the age of 18. Before completing the questionnaire, participants also presented a parental authorization. This study was approved by the school Parents' Association and the school administrators. Due to missing data, one participant had to be excluded from the sample.

### Instruments

#### Resilience

*Escala de Resiliencia para Escolares* (ERE, Resilience Scale for Schoolchildren, Saavedra and Castro, 2009). These Chilean authors developed this instrument to understand how resilience can be a key personal factor to face extreme experiential situations—Chile is considered a leading global example of resilience (Vergara, 2014). Authors pointed out that resilience is a learned trait, and therefore changeable (Varas and Saavedra, 2011). This point of view led Varas and Saavedra (2011) to consider the interactive sources of resilience by following Grotberg's perspective (see Grotberg, 1996, 2003). According to Grotberg (2003), there are three resilience sources: (1) perceived support a person believes he/she has; (2) personal psychological strengths and internal conditions; and (3) problem-solving abilities and skills.

The original scale, designed for adults, was comprised of 60 items regarding 12 resilience factors named identity, autonomy, satisfaction, pragmatism, links, networks, models, goals, affectivity, self-efficacy, learning, and asking for others' assistance (see Saavedra and Castro, 2009). However, Saavedra and Castro (2009) developed a shorten 27-items instrument regarding three resilience factors grouped into *identity-self efficacy* (referring to perceived personal and internal strengths of the children/adolescents); *networks-models* (perceived support from others); and *learning-asking for others' assistance* (perceived ability to solve own and others' problems). Finally, a total score for resilience is also given. Responses were given on a 5-point Likert scale ranging from 1 (*strongly disagree*) to 5 (*strongly agree*). Cronbach's alpha for each of the subscales were 0.55, 0.85, and 0.81 respectively. Reliability for total ERE score was 0.87. Due to the high correlations found between the three subscales of ERE (above 0.75), only total resilience score was used in this study.

#### Emotion regulation ability

Sections D and H of the Mayer-Salovey- Caruso Emotional Intelligence Test, Spanish version adapted to the Spanish context by Extremera et al. (2006) (MSCEIT-S). MSCEIT measures four skill groups of EI: (a) perceiving emotion accurately, (b) using emotion to facilitate thought, (c) understanding emotion, and (d) managing emotion (Grunes et al., 2013). This is a performance test, based on the conceptualization of emotional intelligence as ability, involving problem-solving with and about emotions. For the purpose of this study, only sections regarding management of emotions (sections D and H) were administered to participants. ERA is assessed asking adolescents to evaluate the effectiveness of several actions making an individual feel in a certain way. The questions described different situations (e.g., Debbie just came back from vacation. She was feeling peaceful and content. How well would each action preserve her mood?). Several actions are presented afterwards, (e.g., she started to make a list of things at home that she needed to do) and respondents had to identify the most adaptive ways to regulate own and others' feelings. Responses were given on a Likert scale ranging from 1 (*very ineffective*) to 5 (*very effective*). This section includes six situations with three alternatives to be evaluated out of 18 items.

This test has been chosen due to two main reasons. First, it is based on the emotional intelligence ability model developed by Salovey and Mayer (Lopes et al., 2012). Second, previous studies found limitations regarding self-report measures of emotional intelligence ability such as insufficient reliability and connections with personality factors even in Spanish samples (Extremera et al., 2006). Even though MSCEIT was designed for individuals aged 17 and above, this instrument is used in this study due to the high reliability of the test assessing emotional regulation abilities with high-school adolescents (see Mestre et al., 2006; Lopes et al., 2012). Split-half reliability (corrected using Spearman-Brown formula) for sections D and H of MSCEIT was 0.83.

#### Cognitive emotion regulation strategies

Cognitive Emotion Regulation Questionnaire Spanish version (CERQ-S, Domínguez-Sánchez et al., 2013). The original version was created and validated by Garnefski et al. (2001). CERQ-S is a 36-item questionnaire measuring cognitive emotional regulation strategies used in response to stressful life events. The test consists of nine subscales, with four items per subscale. Responses were given on a 5-point Likert scale ranging from 1 (*almost never*) to 5 (*almost always*). Example items were “I want to understand why I feel the way I do about what I have experienced” and, “I think that I can become a stronger person as a result of what has happened.” The subscales are the following: self-blame, other-blame, acceptance, planning, positive refocusing, rumination or focus on thought, positive reappraisal, putting into perspective, and catastrophizing. Cronbach's alpha for this scale was 0.84. Subscales had reliabilities ranging from 0.53 to 0.74.

#### Control variables: personality traits and verbal intelligence

High School Personality Questionnaire Spanish version (HPSQ-S, Cattell and Cattell, 1986). HSPQ comprised 140 items regarding personality traits of adolescents and their relation with school and social activities. The items are distributed in 14 factors, in which 13 of the 14 factors, measure personality traits and the remaining mental ability or general intelligence. The questionnaire measures primary personality constructs. Factorial research has differentiated the 14 scales, and each of the factors represents different dimensions of personality (Aluja and Blanch, 2004). In this research, we used factors for personality traits: sociability, intelligence, ego-strength, excitability, dominance, enthusiasm, conformity, boldness, sensitivity, withdrawal, apprehension, self-sufficiency, self-discipline, and tension. Participants had to answer several questions with three different choices. Example items were “Do you find it easy to go and introduce yourself to an important person? Yes, maybe, no,” and “what kind of movies do you like best? Musicals, I'm not sure, war.” Cronbach's alpha for the 14 dimensions were ranged from 0.64 to 0.81.

We used a standardized, multi-level test of general intelligence, entitled “Inteligencia General Factorial” (IGF3-R; Yuste, 1997). The test was initially developed in Spanish and has been validated for the Spanish student population. It is a cognitive performance instrument, which measures verbal reasoning, verbal understanding, spatial aptitude, and numerical and abstract reasoning. We used the intermediate version, comprised of 24 items, recommended for high school samples. For the sake of parsimony, we only reported verbal intelligence score in the 13–16-year-old age group. Split-half reliability (corrected by the Spearman-Brown formula) for verbal intelligence IGF3-R subscale was 0.83.

## Results

Before presenting the results of the two primary objectives of the research, descriptive statistics and correlations among all variables involved, are presented in Tables 1, 2. Table 1 also shown correlations between predictors and criterion variable.

**Table 1:** Instruments descriptive statistics and correlations with total resilience.

**Instruments**	**Scales**	***M***	***SD***	**Min–Max**	**Resilience**
	Identity-self efficacy	34.56	4.16	23–45	0.76^***^
	Networks-models	38.18	5.56	17–45	0.89^***^
ERE	Learning-generativity	38.55	4.81	24–45	0.87^***^
	Total resilience	111.29	12.26	75–133	–
MSCEIT	Emotion regulation ability (sections D and H)	88.01	13.45	59.87–130.88	0.39^**^
	Self-blame	11.43	3.11	6–20	0.23^**^
	Acceptance	13.42	2.84	6–20	0.31^**^
	Focus on thought/rumination	13.77	3.09	4–20	0.36^**^
CERQ	Positive refocusing	13.44	4.10	4–20	0.27^**^
	Refocus on planning	14.62	3.48	4–20	0.36^**^
	Positive refocussing	14.38	3.11	4–20	0.36^**^
	Putting into perspective	13.46	3.18	6–20	0.14
	Catastrophizing	10.94	3.19	4–19	0.06
	Blaming others	9.97	2.83	4–18	−0.06
	Sociability	5.19	1.82	1–10	0.23^**^
	Intelligence	3.54	1.88	1–9	0.08
	Ego-strength	5.53	1.86	1–10	0.20^*^
	Excitability	5.15	1.76	1–9	−0.10
	Dominance	6.04	1.95	1–10	−0.21^**^
	Enthusiasm	5.04	1.75	1–9	−0.08
HPSQ-S	Conscientiousness	4.66	1.78	1–9	0.22^**^
	Venturesome	6.03	1.63	1–9	0.21^**^
	Sensitivity	5.62	1.71	2–10	0.09
	Individualism	7.38	1.78	1–10	0.05
	Apprehension	4.63	1.91	1–10	−0.07
	Self-sufficiency	6.06	1.71	1–10	0.01
	Will power	5.39	1.79	1–10	0.20^**^
	Tension	4.90	1.64	1–10	−0.08
IGF3-R	Verbal Intelligence	43.06	15.16	12.50–83.33	0.12

* p < 0.05;

** p < 0.01;

*** *p < 0.001*.

*ERE, Escala de Resiliencia Escolar; MSCEIT, Mayer-Salovey-Caruso Emotional Intelligence Test; CERQ, Cognitive-Emotion Regulation Strategies; HSPQ-S, High School Personality Questionnaire Spanish Version; IGF3-R, Inteligencia General Factorial*.

**Table 2:** Correlations between all variables of the study (*N* = 164).

	**1**	**2**	**3**	**4**	**5**	**6**	**7**	**8**	**9**	**10**	**11**	**12**	**13**	**14**	**15**	**16**	**17**	**18**	**19**	**20**	**21**	**22**	**23**	**24**	**25**	**26**	**27**	**28**	**29**	**30**
1 Total Resilience	*																													
2 Identity-self efficacy	0.76^**^	*																												
3 Networks	0.89^**^	0.50^**^	*																											
4 Learning-generativity	0.87^**^	0.49^**^	68^**^	*																										
5 Age	0.14	0.03	0.15	0.16	*																									
6 Gender (0: males; 1: females)	0.10	−0.01	0.11	0.14	−0.02	*																								
7 ERA (MSCEIT)	0.39^**^	0.29^**^	0.34^**^	0.35^**^	0.08	0.28^**^	*																							
8 IV (IGF)	0.12	0.12	0.09	0.11	−0.05	0.08	0.18^**^	*																						
9 Self-blame (CERQ)	0.23^**^	0.11	0.22^**^	0.23^**^	0.23^**^	−0.07	0.21^**^	−0.11	*																					
10 Acceptance (CERQ)	0.31^**^	0.17^*^	0.29^**^	0.30^**^	0.15	0.04	0.11	0.08	0.42^**^	*																				
11 Focus on thought (CERQ)	0.36^**^	0.16^*^	0.37^**^	0.35^**^	0.16^*^	0.14	0.30^**^	0.03	0.40^**^	0.49^**^	*																			
12 Positive refocusing (CERQ)	0.27^**^	0.20^**^	0.27^**^	0.20^**^	0.17^**^	−0.06	0.00	−0.01	0.17^*^	0.36^**^	0.21^**^	*																		
13 Refocus on planning (CERQ)	0.36^**^	0.27^**^	0.34^**^	0.28^**^	0.16^*^	−0.05	0.26^**^	0.09	0.20^*^	0.25^**^	0.38^**^	0.37^**^	*																	
14 Positive reappraisal (CERQ)	0.36^**^	0.24^**^	0.36^**^	0.31^**^	0.22^**^	−0.07	0.17*	−0.02	0.34^**^	0.33^**^	0.40^**^	0.37^**^	0.60^**^	*																
15 Perspective (CERQ)	0.14	0.06	0.16^*^	0.12	0.12	0.07	0.11	0.05	0.29^**^	0.21^**^	0.24^**^	0.27^**^	0.25^**^	0.41^**^	*															
16 Catastroohizing (CERQ)	0.06	−0.01	0.04	0.10	0.08	0.09	−0.04	−0.14	0.28^**^	0.23^**^	0.20^*^	−0.08	0.04	0.06	0.25^**^	*														
17 Blaming others (CERQ)	−0.06	0.04	−0.11	−0.05	−0.04	−0.27^**^	−0.19^*^	−0.22^**^	0.12	0.06	0.07	0.12	0.13	0.04	0.07	0.40^**^	*													
18 Sociability (HSPQ) [A]	0.23^**^	0.26^**^	0.14	0.20^**^	−0.02	−0.09	0.01	0.14	−0.05	0.13	0.02	0.04	0.16*	0.05	−0.08	−0.04	−0.05	*												
19 Intelligence (HSPQ) [B]	0.08	0.09	0.03	0.09	−0.19^*^	−0.02	0.12	0.38^**^	0.00	0.04	0.09	−0.07	0.12	0.02	−0.03	0.06	−0.10	0.06	*											
20 Ego-strength (HSPQ) [C]	0.20^**^	0.31^**^	0.16^*^	0.05	−0.09	−0.09	0.06	−0.02	−0.13	0.03	−0.04	0.25^**^	0.14	0.16^*^	−0.03	−0.21^**^	−0.01	0.08	−0.02	*										
21 Excitability (HSPQ) [D]	−0.10	−0.11	−0.12	−0.03	0.03	0.01	−0.11	0.10	0.08	−0.12	−0.02	−0.15	−0.06	0.00	0.02	0.11	0.09	0.04	−0.01	−0.33^**^	*									
22 Dominance (HSPQ) [E]	0.21^**^	−0.00	−0.27^**^	−0.23^**^	−0.18^**^	−0.09	−0.13	0.06	−0.26^**^	0.00	−0.26^**^	−0.04	−0.08	−0.14	−0.10	−0.07	0.04	−0.06	0.02	0.11	−0.05	*								
23 Enthusiasm (HSPQ) [F]	−0.08	0.00	−0.09	−0.10	0.09	0.05	−0.05	0.06	0.03	0.03	−0.10	−0.08	−0.01	−0.07	−0.14	0.13	−0.02	0.04	−0.02	0.09	0.06	0.21^**^	*							
24 Consciousness (HSPQ) [G]	0.22^**^	0.29^**^	−0.19^*^	0.09	−0.06	−0.05	0.11	0.09	0.04	−0.13	0.05	0.15	0.13	0.08	0.04	−0.07	−0.07	0.11	0.19^*^	0.35^**^	−0.11	−0.14	−0.22^**^	*						
25 Venturesome (HSPQ) [H]	0.21^**^	0.24^**^	0.14	0.16^*^	−0.05	0.19^*^	0.11	0.18^*^	−0.14	0.09	0.06	0.01	0.13	0.05	−0.00	−0.00	0.09	0.27^**^	0.13	0.24^**^	−0.22	0.02	0.02	0.14	*					
26 Sensivity (HSPQ) [I]	0.09	0.05	0.07	0.11	0.01	0.03	0.11	0.02	0.17^*^	0.05	0.08	−0.06	0.12	0.12	0.09	0.14	−0.09	0.04	0.14	−0.11	0.07	−0.28^**^	−0.09	0.05	−0.07	*				
27 Individualism (HSPQ) [J]	0.04	−0.01	0.09	0.02	0.16^**^	0.38^**^	0.18^*^	−0.02	0.06	−0.01	0.13	0.01	0.02	0.07	0.14	0.06	−0.15	−0.16^*^	−0.04	−0.16^*^	0.08	−0.16^*^	0.01	0.07	−0.11	0.07	*			
28 Apprehension (HSPQ) [O]	−0.07	−0.21^**^	0.03	−0.03	0.07	0.24^**^	0.09	−0.01	0.12	0.02	0.10	−0.17	−0.18*	−0.09	0.08	0.10	−0.09	−0.19^*^	−0.09	−0.31^**^	0.11	−0.29^**^	−0.07	−0.03	−0.15	0.01	0.16^*^	*		
29 Self-sufficiency (HSPQ) [Q2]	0.01	−0.05	0.07	−0.01	−0.00	0.05	0.09	−0.08	0.03	−0.05	0.00	0.00	−0.08	−0.03	0.05	−0.04	−0.07	−0.34^**^	−0.06	−0.06	−0.05	−0.06	−0.23^**^	0.04	−0.28^**^	0.03	0.14	0.13	*	
30 Willpower (HSPQ) [Q3]	0.20^**^	0.19^*^	0.21^**^	−0.01	−0.00	0.04	0.11	03	−0.13	−0.09	−0.00	0.14	0.13	0.12	0.14	−0.14	−0.08	0.13	0.04	0.33^**^	−0.33^**^	−0.19^*^	−0.30^**^	0.41^**^	−0.23^**^	0.07	0.01	−0.14	0.05	*
31 Tension (HSPQ) [Q4]	−0.08	−0.14	0.11	0.09	0.09	−0.04	−0.02	−0.13	0.10	−0.02	0.02	−0.23^**^	−0.03	−0.14	−0.03	0.25^**^	0.10	−0.03	−0.03	−0.39^**^	0.26^**^	0.05	−0.07	−0.09	−0.16^*^	0.13	0.13	0.16^*^	−0.09	−0.30^**^

*In bold significative correlations*.

* p < 0.05;

** *p < 0.01. MSCEIT, Mayer-Salovey-Caruso Emotional Intelligence Test; CERQ, Cognitive-Emotion Regulation Strategies; HSPQ-S, High School Personality Questionnaire Spanish Version; IGF3-R, Inteligencia General Factorial*.

Predictor variables should be selected taking into consideration previous research or pilot studies that may have guided the introduction of these predictors. Due to the lack of studies including ERA and resilience, the inclusion of these variables must be guided purely by mathematical criteria. In our study we have used a backward method, following Field's suggestion: “If you do decide to use a stepwise method then the backward method is preferable to the forward method. This is because of suppressor effects, which occur when a predictor has a significant effect but only when another variable is held constant. Forward selection is more likely than backward elimination to exclude predictors involved in suppressor effects. As such, the forward method runs a higher risk of making a Type II error (i.e., missing a predictor that does in fact predict the outcome)” (Field, 2013, p. 213).

First, Table 2 (correlations) was studied, and then regression analyses were performed, in order to find the variables that better predicted Total Resilience (dependent variable). Finally, was found that these variables were ERA (MSCEIT), Positive reappraisal (CERQ), and Sociability (HSPQ).

In addition to the analysis of the complete sample, a cross-validation study was performed, in order to avoid, as far as possible, the “over-fitting” effect of the model (including too many variables) or “under-fitting” (leaving out predictors that actually matter), when performing a stepwise regression. Descriptive statistics for total sample and both halves and are presented in Table 3. Although sample size may be problematic, such a study is strongly recommended in exploratory models (Field, 2013). Taking into consideration the 80% of the sample, the model shows a good adjustment, representing 31.0% of variance explained of the dependent variable (Table 4).

**Table 3:** Descriptives for each sample analysis (total, 80%, and 20%) for the cross-validation study.

**Sample**	**Total (*N* = 164)**	**Random 80% (*n* = 129)**	**Random 20% (*n* = 32)**
	***M* (*SD*)**	***M* (*SD*)**	***M* (*SD*)**
Total Resilience (ERE)	111.29 (12.26)	111.35 (12.45)	111.18 (11.73)
ERA (MSCEIT)	88.01 (13.45)	88.24 (13.35)	85.87 (11.70)
Positive Reappraisal (CERQ)	14.38 (3.11)	14.33 (3.12)	13.77 (3.70)
Sociability (A factor, HSPQ)	5.19 (1.82)	5.33 (1.83)	5.09 (1.95)

*ERE, Escala de Resiliencia Escolar (School Resilience Scale); MSCEIT, Mayer-Salovey-Caruso Emotional Intelligence Test; CERQ, Cognitive-Emotion Regulation Questionnaire; HSPQ-S, High School Personality Questionnaire*.

**Table 4:** Coefficients of backward regressions, 95% Interval of confidence, zero-order correlations, and variance inflation factors of the cross-validation (total, 80%, and 20% of the sample).

		**Values presented for Total (In parenthesis values for 80%) [In brackets values for 20%]**			
		**(Constant)**	**Emotion regulation ability MSCEIT**	**Positive reappraisal CERQ**	**Sociability [A] HSPQ**
Unstandardized coefficients	B	60.25 (61.74) [64.09]	0.31 (0.28) [0.27]	1.16 (1.32) [1.54]	1.41 (1.10) [0.48]
	Std. Error	6.51 (7.23) [12.43]	0.06 (0.07) [0.13]	0.27 (0.30) [0.41]	0.45 (0.49) [0.77]
Standardized coefficients	Beta		0.34 (0.30) [0.27]	0.29 (0.33) [0.48]	0.21 (0.16) [0.08]
	t	9.25 (8.54) [5.15]	4.96 (4.05) [2.11]	4.34 (4.43) [3.80]	3.12 (2.22) [0.63]
	Sig.	0.000 (0.000) [0.000]	0.000 (0.000) [0.041]	0.000 (0.000) [0.000]	0.002 (0.028) [0.535]
95.0% Confidence Interval for B	Lower Bound	43.9 (47.44) [38.96]	0.18 (0.14) [0.01]	0.63 (0.73) [0.72]	0.52 (0.12) [−1.08]
	Upper Bound	73.12 (76.03) [89.21]	0.43 (0.42) [0.53]	1.69 (1.91) [2.37]	2.30 (2.08) [2.04]
Correlations	Zero-order		0.39 (0.36) [0.32]	0.36 (0.41) [0.51]	0.23 (0.18) [0.12]
Multicollinearity	Tolerance		0.97 (0.96) [0.99]	0.97 (0.95) [0.99]	0.99 (0.99) [0.99]
Statistics	VIF		1.03 (1.04) [1.01]	1.03 (1.05) [1.01]	1.00 (1.01) [1.00]

*Total resilience (DV). VIF, Variance Inflation Factors*.

Regarding the control of multicollinearity, measures of variance inflation factors (VIF) are provided in Table 4. VIF measures are below the recommended values of tolerance. Therefore, no problems of multicollinearity are observed. Moreover, all tolerance values are well above 0.2 (Bowerman and O'Connell, 1990), and the correlations matrix does not present any value high enough to confront the results.

After controlling for multicollinearity stepwise multiple regression analysis was performed (Table 5).

**Table 5:** Multiple stepwise regression analysis of predictors of self-reported resilience.

**Variable**	**Self-report resilience**				
	**Model 1 *B***	**Model 2**		**Model 3**	
		***B***	***B***		**95% CI**
Constant	80.06^**^	67.01^**^	60.25^**^		[47.39, 73.12]
ERA (MSCEIT)	0.39^**^	0.34^**^	0.34^**^		[0.19, 0.43]
Positive reappraisal (CERQ)		0.31^**^	0.30^**^		[0.63, 1.70]
Sociability (HSPQ-S)			0.21^**^		[0.52, 2.30]
*R*^2^	0.15	0.24		0.29	
*F*	28.93^**^	25.71^**^		21.36^**^	
*ΔR*^2^		0.91		0.04	
*ΔF*		19.23		9.83	

N = 164. ^*^p < 0.05;

** *p < 0.01. CI, confidence interval; MSCEIT, Mayer-Salovey-Caruso Emotional Intelligence Test; CERQ, Cognitive-Emotion Regulation Strategies; HSPQ-S, High School Personality Questionnaire Spanish Version*.

## Discussion

This research aimed to assess the role of emotional-cognitive abilities on resilience perception of adolescents from a suburban area. Literature up to date has been focused on the relationship between resilience and psychosocial factors (Hunter and Chandler, 1999; Jenson and Fraser, 2005; Ungar et al., 2005, 2014; Tusaie et al., 2007). However, little is known about the relationship between emotional and cognitive skills and resilience among young people.

Regarding the resilience scale employed, we decided to use a truthful instrument for Spanish samples instead of other measures developed under different cultural meanings (f. i., Von Soest et al., 2010). ERE (Saavedra and Castro, 2009) was validated among cross-cultural Spanish-speaking adolescent samples. In our study, we found similar descriptive measures (*M* = 111.29, *SD* = 12.26, *N* = 164), than those obtained by Saavedra and Castro (2009) (*M* = 116.2, *SD* = 11, *N* = 300) and Hewitt et al. (2014) (*M* = 110.24, *SD* = 21.31, *N* = 289) with Chilean and Colombian adolescents respectively. However, in the study developed by González-Arratia López Fuentes et al. (2008, 2012), with Mexican participants, total ERE scores were higher than in those cited before (*M* = 131.9, *SD* = 12.4, *N* = 300). Despite cultural and idiosyncratic differences, our Spanish sample obtained similar descriptive results. Although all samples were collected from suburban and risky areas, these studies have shown that adolescents tend to perceive themselves as moderate-strong resilient.

Risk factors and vulnerabilities are predisposing elements that may influence individual's healthy behavior, especially under unfavorable environments (Vanderbilt-Adriance and Shaw, 2008; Grant and Kinman, 2012). Adolescents need to face these adversities to successfully adapt to the environment. Hence, what factors influence the development of resilience?

On the one hand, the environment provides its own protective factors represented in figures, resources, or appropriate contexts for a healthy individual development. In this line of reasoning, Beddoe et al. (2013) suggested to include social work education for preparing resilient practitioners. On the other hand, several studies have pointed out how individual differences can explain the use of individual strategies to successfully overcome adversity. For instance, Nota et al. (2004) found out that some aspects of personality, translated into different degrees of vulnerability, can facilitate the emergence of modulators in the resilient process.

Our sample shared similar environmental influences; henceforth, psychological factors should explain the differences in perceived resilience among adolescents. Our results revealed moderate to high levels of total resilience among teenagers, which means that sample adolescents perceive themselves able to overcome this risky environment and to adapt in the face of adversity (Lee et al., 2013). These results are in line with previous research, which stated that children under low socioeconomic environments develop high levels of resilience helping them to successfully adapt to stress (Wang and Gordon, 1994).

Mean ERA score (*M* = 88.01, *SD* = 13.45) yielded low levels of managing emotion for normative samples. Note that MSCEIT scores computed by test publishers are standardized (*M* = 100, *SD* = 15) (Mayer et al., 2003; Roberts et al., 2006; Karim and Weisz, 2010). ERA subscale of MSCEIT (Spanish version by Extremera et al., 2006), was validated in a sample of 946 Spanish individuals obtaining lower mean ERA scores than in the original study (*M* = 96.31, *SD* = 11.54). Due to cultural biases (Extremera et al., 2006; Karim and Weisz, 2010) and sample average age, mean ERA score was as expected and similar to other studies using Spanish adolescents (Mestre et al., 2006; Lopes et al., 2012). These results imply that at this life stage, adolescents have not completely developed ERA (Zeidner et al., 2003). Probably because this is the most complicated ability to acquire within the Mayer and Salovey's emotional intelligence model (Mayer and Salovey, 1997; Zeidner et al., 2003; Dulewicz and Higgs, 2004; Gratz and Roemer, 2004; Urry and Gross, 2010; Côté et al., 2011).

Due to the multicollineality values obtained in Table 4, no suppressor effect is found in the data. Moreover, comparing beta coefficients between both portions (80 and 20%), they are statistically alike. Matching determination coefficients, they are very similar, 0.27 and 0.34; also analogous to the *R*^2^ = 0.28 using the whole sample. We can conclude that, despite very limited sample size (affecting mostly 20% half) the model can be cross-validated.

To verify the predictive capability of the model, a stepwise multiple regression analysis was performed. Only variables, which significantly correlated with resilience, were included. According to the coefficient of determination of model 1 (*R*^2^ = 0.15, *p* < 0.01), ERA explained 15% of the variance of perceived resilience among adolescents. ERA involves the capacity to modulate feelings to promote personal understanding and growth (Mayer and Salovey, 1997). Therefore, adolescents with higher ERA scores exhibited higher perceived resilience levels. These results involve that adolescents with high scores on ERA, may show better outcomes in mental health, displaying higher levels of well-being and lower depression symptoms (Fernandez-Berrocal and Extremera, 2016), may have better job opportunities (Lopes, 2016), and may also reach better standards for school adaptation (Rivers et al., 2012). Other studies using MSCEIT measures (especially sections D and H, managing emotions) have also found associations between ERA and resilience. For instance, Schneider et al. (2013) found that emotional intelligence facilitates stress resilience. Frajo-Apor et al. (2016) examined emotional intelligence and resilience concerning the mental health of first-year college students finding positive correlations between both variables. Caston and Mauss (2011) also found that ERA is a protective factor that promotes a resilient response when facing stressful stimuli. Even though none of these studies used adolescents as participants, our study revealed similar findings. As ERA can be taught (Barchard et al., 2016; Nathanson et al., 2016) it is essential to keep in mind that fostering adolescents' ability to regulate own and other's emotions can improve their real-life management of emotions (Peña-Sarrionandia et al., 2015). In this sense, by promoting the ability to manage emotions, adolescents will perceive themselves able to adapt in the face of adversity. These results are in line with Montgomery et al. (2008), who found a relationship between both variables resilience, and ERA, in young adults with Asperger's disorder. Even though, our sample is not officially diagnosed with any mental illness, we consider that the environment is crucial for a child's development. For that reason, adolescents growing under a risk context (i.e., low socioeconomic environment) can face emotional and psychological difficulties that, in turn, can undermine their mental health (Ng et al., 2012). As mention in the introduction section, literature is mainly focused on the relationship between emotional intelligence and resilience, in mental illness patients (Frajo-Apor et al., 2016; Artuch-Garde et al., 2017), but little is known about the role played by these two variables in healthy youths. In this sense, our article contributes to prior research increasing previous knowledge in a non-psychopathological sample.

Regarding *coping*, mean CERQ dimensions results (see Table 1) are similar to those obtained by Domínguez-Sánchez et al. (2013) in their Spanish validation of the instrument. Correlation analysis between all variables included in the study revealed significant positive correlations between resilience and ERA, self-blame, acceptance, focus on thought/rumination, positive refocusing, refocus on planning, positive reappraisal, sociability, ego-strength, conscientiousness, venturesome, and willpower. Moreover, correlation analysis also showed a significant negative correlation between resilience and dominance.

After including the emotional-cognitive strategy, *positive reappraisal*, our model increases its predictivity in 9%. According to these results, promoting adolescents' ability to positively reinterpret stressful situations can also enhance their resilience perception. Positive reappraisal is a coping strategy typically related to resilience (Bonanno and Mancini, 2008). Min et al. (2013) found that this strategy is associated with resilience, in individuals diagnosed with anxiety and/or depression. Furthermore, research has shown that other cognitive coping strategies are related to resilience levels. For instance, positive coping strategies, such problem-focused coping, infusing positive meaning to ordinary life events, and once again positive reappraisal are associated with the occurrence and maintenance of positive affect (Folkman and Moskowitz, 2000), ultimately predicting increases in psychological well-being and health (Affleck and Tennen, 1996). Coping has also been associated with negative emotions (anxiety, anger), aggression (Ng et al., 2012), and secure attachment (Li, 2008).

As mention before, people use positive emotions to recover from stressful situations (Fredrickson et al., 2000; Tugade and Fredrickson, 2004). Indeed, earlier theoretical writings have indicated that resilient individuals are characterized by high positive emotionality (Block and Kremen, 1996). In order to enhance positive emotions, resilient individuals use positive reappraisal to extract positive meaning from adverse circumstances (Garnefski et al., 2001; Tugade and Fredrickson, 2004; Ong et al., 2006; Caston and Mauss, 2011; Ng et al., 2012; Min et al., 2013; Kalisch et al., 2015; Fernandez-Berrocal and Extremera, 2016; Hughes and Evans, 2016; Mestre et al., 2016). Therefore, this individual factor appears to be a key element developing resilience in different environments. According to the above mention, teaching mindfulness can be an exciting way to promote this vital coping strategy (Garland et al., 2009). Moreover, and considering resilience is largely associated with well-being (Craciun, 2013), fostering both, ERA and positive cognitive-regulation strategies in intervention programs can also help adolescents to develop resilient outcomes. It is important to note that adaption to stressful stimuli at this life stage is crucial (Ong et al., 2006; Kinman and Grant, 2011), and can determine adolescent adjustment in their forthcoming future (Feragen et al., 2009).

To a lesser extent, *sociability* (A factor of HSPQ) also contributes to a better resilience perception among adolescents (ΔR^2^ = 0.04). Including all predictive variables, sociability, ERA, and positive reappraisal, our model shows that these abilities are good predictors of efficient social functioning (Eisenberg et al., 2004). As shown in Table 2, the relationship between ERA, cognitive-emotional regulation strategies, and sociability explained 29% of the variance of perceived resilience among adolescents. In line with our findings, Hopp et al. (2011) found that people who exhibit a resilient outcome after a stressor are less vulnerable to be affected by negative outcomes. Hence, these results also support our view of resilience, in the sense that adverse situations and environmental interactions are necessarily for developing a resilient response.

The resilience scale used in this study (ERE), has been applied and validated in countries with similar characteristics to the Spanish culture. However, we obtained high correlations between ERE subscales showing dependency between its different elements. Furthermore, we could not determine whether ERA was related or not to other factors of resilience such as generativity, or personal strengths. Although we only considered one ERE factor (total resilience score), our study is congruent with the meta-analysis carried out by Lee et al. (2013). In their study, the authors pointed out that correlations between risk factors and resilience were less significant than correlations between protective factors and resilience. Positive affectivity, self-efficacy, and self-esteem appeared as the most important protective factors promoting resilience.

In our study, verbal intelligence, most of the personality traits, gender, and age were not significantly related to resilience. Hence, our research stresses the importance of ERA explaining perceived resilience among adolescents. As Wang et al. (1995) pointed out, protective factors depend on the context. ERA, coping, and sociability may be related to adolescent resilience, but not necessarily in all contexts.

Our research presents a few limitations. The first of all is related to the resilience concept. Whether resilience is a personality trait or a set of developmental process, there is not a strong theoretical background that determines how a researcher should evaluate this topic. To overcome this difficulty, it will be necessary to conduct longitudinal and qualitative studies to deeply understand how the environment and people's development evoke resilient responses. A second limitation refers to ERE subscales. Although we have similar average resilience scores as other countries have (Chile and Colombia), ERE subscales did not show independency. Hence, this measure did not allow to assess the relationships between predictive variables and ERE's resilience subscales. Another limitation is the sample size. Some of the predictive variables would result significant predictors of resilience by increasing the sample. Moreover a larger sample size would also provide more reliable estimates to the effects. Finally, the reliability of some subscales of CERQ is questionable. This instrument might be used following the recommendations of Domínguez-Sánchez et al. (2013), who found two-second order factors (synergistic and antagonistic strategies).

## Implications

Traditional research, including MSCEIT measures, has found differences regarding gender, where women have usually obtained higher scores than men (Extremera et al., 2006; Mestre et al., 2006). One of the advantages of this sample was its heterogeneity because we did not find gender differences. One of the reasons may be the use of high-school adolescents (without educational filters) as participants, instead of undergraduate students. During Spanish compulsory schooling, both emotional, and cognitive competencies can be taught and, hence, these protective factors can result in resilient behaviors.

Under educational resilience umbrella (see Randolph et al., 2004), adolescents develop protective factors to prevent stressful situations and adverse environments. One of these protective factors is ERA. Our findings support the increasing implementation of training programs in social-emotional-learning activities, focused on increasing ERA (Keller and Otto, 2009; Rivers et al., 2012; Nathanson et al., 2016). In this line of reasoning, teaching ERA during compulsory schooling can foster healthy skills to help adolescents facing adverse circumstances in their forthcoming future.

## Ethics statement

To protect confidentiality of participants, this research was conducted following the APA Ethics Code Standard 3.08. Besides, the study was approved by the Ethical Board of the Universidad de Cádiz and the high-school participants had to present a signed consent of their parents or legal tutors (to be included in the study).

## Author contributions

JM, JN-L, and RG-M led the research and the whole process of writing the paper. JN-L conducted the research under JM and RG supervision, who designed the research and contributed in a previous pilot study. RG and AZ made substantial contributions about data analysis and interpretation of the findings. JM and RG-M wrote the paper under final supervision of both RG and AZ.

### Conflict of interest statement

The authors declare that the research was conducted in the absence of any commercial or financial relationships that could be construed as a potential conflict of interest.
